# CRISPR/Cas9-derived models of ovarian high grade serous carcinoma targeting *Brca1*, *Pten* and *Nf1*, and correlation with platinum sensitivity

**DOI:** 10.1038/s41598-017-17119-1

**Published:** 2017-12-04

**Authors:** Josephine B. Walton, Malcolm Farquharson, Susan Mason, Jennifer Port, Bjorn Kruspig, Suzanne Dowson, David Stevenson, Daniel Murphy, Martin Matzuk, Jaeyeon Kim, Seth Coffelt, Karen Blyth, Iain A. McNeish

**Affiliations:** 1 Institute of Cancer Sciences, University of Glasgow, Glasgow, UK; 2 Cancer Research UK Beatson Institute, Glasgow, UK; 3 Department of Pathology and Immunology, Baylor College of Medicine, Houston, TX USA; 4 Departments of Biochemistry and Molecular Biology, Indiana University School of Medicine, Indianapolis, IN USA

## Abstract

Transplantable murine models of ovarian high grade serous carcinoma (HGSC) remain an important research tool. We previously showed that ID8, a widely-used syngeneic model of ovarian cancer, lacked any of the frequent mutations in HGSC, and used CRISPR/Cas9 gene editing to generate derivatives with deletions in *Trp53* and *Brca2*. Here we have used one ID8 *Trp53* ^−/−^ clone to generate further mutants, with additional mutations in *Brca1*, *Pten* and *Nf1*, all of which are frequently mutated or deleted in HGSC. We have also generated clones with triple deletions in *Trp53*, *Brca2* and *Pten*. We show that ID8 *Trp53* ^−/−^;*Brca1* ^−/−^ and *Trp53* ^−/−^;*Brca2* ^−/−^ cells have defective homologous recombination and increased sensitivity to both platinum and PARP inhibitor chemotherapy compared to *Trp53* ^−/−^. By contrast, loss of *Pten* or *Nf1* increases growth rate *in vivo*, and reduces survival following cisplatin chemotherapy *in vivo*. Finally, we have also targeted *Trp53* in cells isolated from a previous transgenic murine fallopian tube carcinoma model, and confirmed that loss of p53 expression in this second model accelerates intraperitoneal growth. Together, these CRISPR-generated models represent a new and simple tool to investigate the biology of HGSC, and the ID8 cell lines are freely available to researchers.

## Introduction

HGSC is the commonest subtype of epithelial ovarian cancer and accounts for approximately 80% of ovarian cancer deaths. It is marked by universal *TP53* mutation^1^ and extreme genomic instability^2,3^. Approximately 20% HGSC harbour mutations in *BRCA1* or *BRCA2* ^4^, whilst loss of NF1 and PTEN expression is seen in around 20% cases, largely resulting from complex genomic rearrangements and structural variation^5^. Importantly, the complexity of HGSC means that multiple genomic abnormalities can co-exist within individual tumours^5^. Although robust prognostic and predictive molecular classifiers of HGSC remain elusive, patients with germline *BRCA1* and *BRCA2* mutations have improved overall prognosis^6^ with improved response to platinum^7^ and PARP inhibitor^8^ chemotherapy. Conversely, PTEN loss is a marker of poor prognosis^9^.

Lack of reliable immunocompetent murine models has significantly impeded HGSC research^10^. Recently, new genetically engineered mouse models (GEMM) of HGSC have been developed^11,12^, in which *Trp53*, *Brca1*, *Brca2*, *Pten* and *Nf1* have been deleted in fallopian tube epithelial cells using Cre-mediated recombination. These models have great potential to expand our understanding of HGSC biology, but still require large-scale breeding programmes, and the mice can take many months to develop tumours. Thus, transplantable models remain valuable research tools.

First described in 2000^13^, the ID8 model is a widely-used syngeneic model of ovarian cancer. However, using whole-exome sequencing, we recently showed that ID8 lacked any mutations fundamental in HGSC biology^14^: ID8 cells were *Trp53* wild-type and retained functional p53 signalling. *Brca1* and *Brca2* were both wild-type and ID8 displayed competent homologous recombination DNA double strand break repair. Using CRISPR/Cas9 gene editing, we generated four *Trp53* ^−/−^ and two *Trp53* ^−/−^;*Brca2* ^−/−^ ID8 clones and characterised their intraperitoneal growth^14^.

We have now targeted three more genes critical in HGSC, *Brca1*, *Pten* and *Nf1*, in ID8 *Trp53* ^−/−^ cells, and have also generated lines with triple deletion in *Trp53*, *Brca2* and *Pten*. We have evaluated sensitivity to platinum and PARP inhibitor therapy in these cells, as well as our original *Trp53* ^−/−^ and *Trp53* ^−/−^;*Brca2* ^−/−^ cells. We have also targeted *Trp53* in cells isolated from a previous transgenic murine fallopian tube carcinoma model^15^, in which Cre recombinase, under the control of the anti-Mullerian hormone type 2 receptor promoter (*Amhr2-Cre*), was used to delete *Dicer*, a key processor of microRNAs, and *Pten* selectively in the fallopian tube.

Our results indicate that loss of NF1 or PTEN expression increases intraperitoneal growth in ID8 cells, and is associated with a poor outcome following platinum chemotherapy. The utility of these ID8 derivatives is confirmed by increased sensitivity to both PARP inhibitor and platinum chemotherapy upon loss of BRCA1 or BRCA2 function. In the fallopian tube *Dicer*;*Pten* double knockout (DKO) cells, loss of p53 significantly increases intraperitoneal growth.

## Results

### CRISPR/Cas9 *Brca1*, *Nf1* and *Pten* editing in ID8 cells

**Figure 1:**
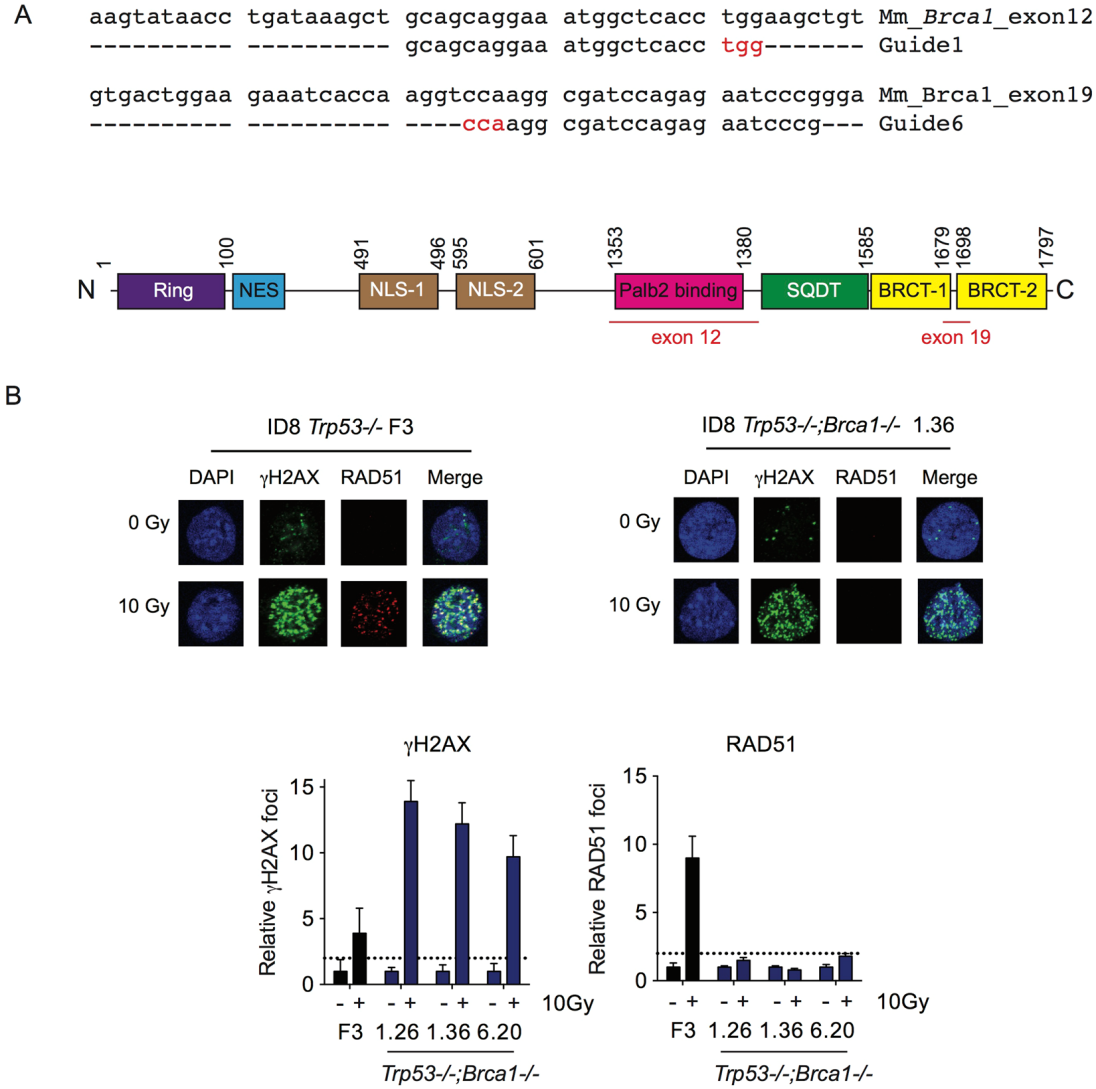
Generation of *Trp53* ^−/−^;*Brca1* ^−/−^ ID8 cells. (**A**) Design of guide RNA targeted to exons 12 and 19 of *Brca1*. Nucleotides in red represent PAM sequence. Schematic representation of BRCA1 protein with exons 12 and 19 highlighted in red (bottom). Numbers represent amino acid positions. (**B**) ID8 *Trp53* ^−/−^ and *Trp53* ^−/−^;*Brca1* ^−/−^ cells were irradiated (10 Gy), fixed and stained for γH2AX and RAD51, and counterstained with DAPI. RAD51 foci were counted in up to 30 untreated and irradiated cells. Bars represent mean (+/− SEM) γH2AX (left) and RAD51 (right) foci per cell; dotted lines represents two-fold increase in γH2AX and RAD51 foci/cell relative to untreated cells, suggestive of induction of DNA double strand breaks and functional homologous recombination respectively^16^.

We used one ID8 *Trp53* ^−/−^ clone (F3)^14^ to generate new sublines with deletions in *Brca1*, *Pten* and *Nf1*. We first targeted the *Brca1* PALB2-binding domain in exon 12 (guide 1) and the BRCT-2 domain in exon 19 (guide 6) (Fig. 1A). Three single cell clones with bi-allelic deletions were isolated following transfection with the two separate guide RNA (clones 1.26, 1.36 - guide 1; clone 6.20 - guide 6; Fig. S1). All three clones failed to generate RAD51 foci in response to 10 Gy irradiation (Fig. 1B) or 10 µM rucaparib (Fig. S2), thus fulfilling criteria for defective homologous recombination (HR)^16^.

**Figure 2:**
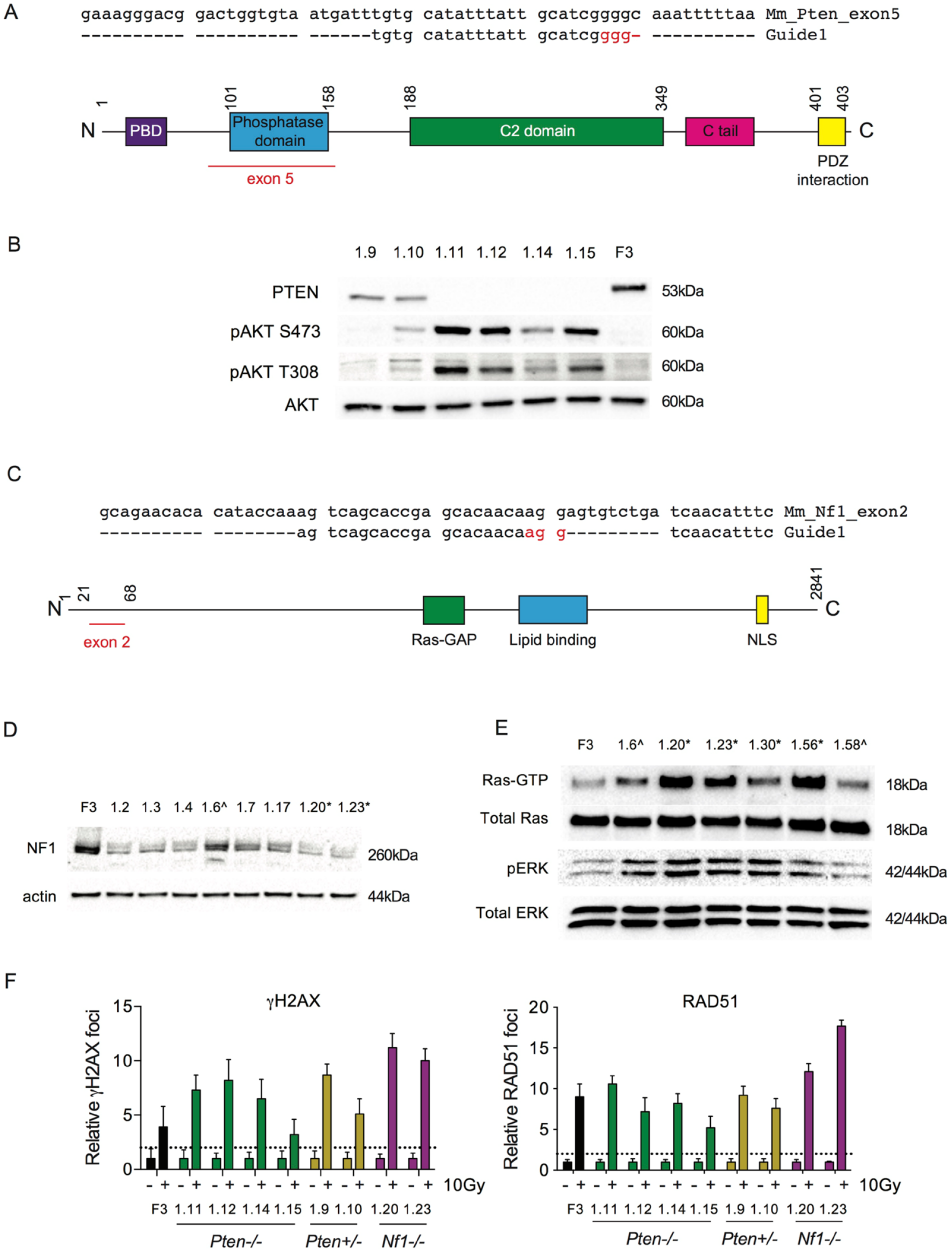
Generation of *Trp53* ^−/−^;*Nf1* ^−/−^ and *Trp53* ^−/−^;*Pten* ^−/−^ ID8 cells. (**A**) Design of guide RNA targeted to exon 5 of *Pten*. Nucleotides in red represent PAM sequence. Schematic representation of PTEN protein with exon 5 highlighted in red (bottom). Numbers represent amino acid position. (**B**) Immunoblot for PTEN and phospho-AKT in clones following *Pten* gRNA transfection. Clones 1.11, 1.12, 1.14 and 1.15 had bi-allelic *Pten* deletions and showed absent PTEN protein with phosphorylation of AKT at both S473 and T308 following serum starvation. Clones 1.9 and 1.10 had mono-allelic deletions. F3 = ID8 *Trp53* ^−/−^. (**C**) Design of guide RNA targeted to exon 2 of *Nf1* as previously^29^. Nucleotides in red represent PAM sequence. Schematic representation of Nf1 protein with exon 2 highlighted in red (bottom). Numbers represent amino acid positions. (**D**) Immunoblot for Nf1 in clones following Nf1 gRNA transfection. *; clones 1.20 and 1.23 had bi-allelic indels confirmed by Sanger sequencing, ^; clone 1.6 had single allele deletion. Clones 1.2, 1.3, 1.4, 1.7 and 1.17 were not sequenced. (**E**) Ras-GTP co-immunoprecipitation and phospho-ERK immunoblot in ID8 clones following Nf1 gRNA transfection. *; clones 1.20, 1.23, 1.30 and 1.56 had confirmed bi-allelic indels and demonstrated increased Ras-GTP pulldown and ERK phosphorylation suggestive of activated Ras signalling. ^; clones 1.6 and 1.58 had single allele deletions. (**F**) ID8 *Trp53* ^−/−^;*Pten* ^−/−^, *Trp53* ^−/−^;*Nf1* ^−/−^ and *Trp53* ^−/−^;*Pten* ^+*/−*^ cells were irradiated (10 Gy), fixed and stained for γH2AX and RAD51, and counterstained with DAPI. RAD51 foci were counted in up to 30 untreated and irradiated cells. Bars represent foci per cell (mean +/− SEM); γH2AX (left) and RAD51 (right); dotted lines represents two-fold increase in γH2AX and RAD51 foci/cell relative to untreated cells as above.

We targeted exon 5 of *Pten*, encoding the phosphatase domain (Fig. 2A). Four clones with bi-allelic deletions (clones 1.11, 1.12, 1.14, 1.15) and two with single allele deletions (clones 1.9, 1.10) were isolated - deletions ranged from 2 bp to >400 bp (Fig. S3). Immunoblots showed absent PTEN in *Trp53* ^−/−^;*Pten* ^−/−^ cells compared to F3, with reduced levels in the heterozygote clones (Fig. 2B). Bi-allelic *Pten* loss resulted in marked increases in AKT phosphorylation at both S473 and T308 upon serum starvation (Fig. 2B). AKT phosphorylation was less marked in one of the heterozygote clones (1.10) than in *Trp53* ^−/−^;*Pten* ^−/−^, although still greater than in F3 control.

For *Nf1*, we targeted exon 2 (Fig. 2C), and used a combination of NF1 expression on immunoblot (Fig. 2D), Raf-RBD co-immunoprecipitation and ERK phosphorylation (Fig. 2E) to screen clones. Clones 1.20, 1.23, 1.30 and 1.56 all had confirmed bi-allelic deletions on Sanger sequencing, whilst 1.6 and 1.58 had single allele changes. We selected two clones (1.20 and 1.23; see Fig. S4 for sequencing data) for further evaluation as they demonstrated increased GTP-bound RAS compared to F3, indicative of activated RAS signalling, as well as increased ERK phosphorylation on immunoblot (Fig. 2E). All *Trp53* ^−/−^;*Nf1* ^−/−^, *Trp53* ^−/−^;*Pten* ^−/−^ and *Trp53* ^−/−^;*Pten* ^+*/−*^ cells were HR competent in 10 Gy irradiation experiments (Figs 2F and S5).

### Generation of triple-deleted ID8 lines

**Figure 3:**
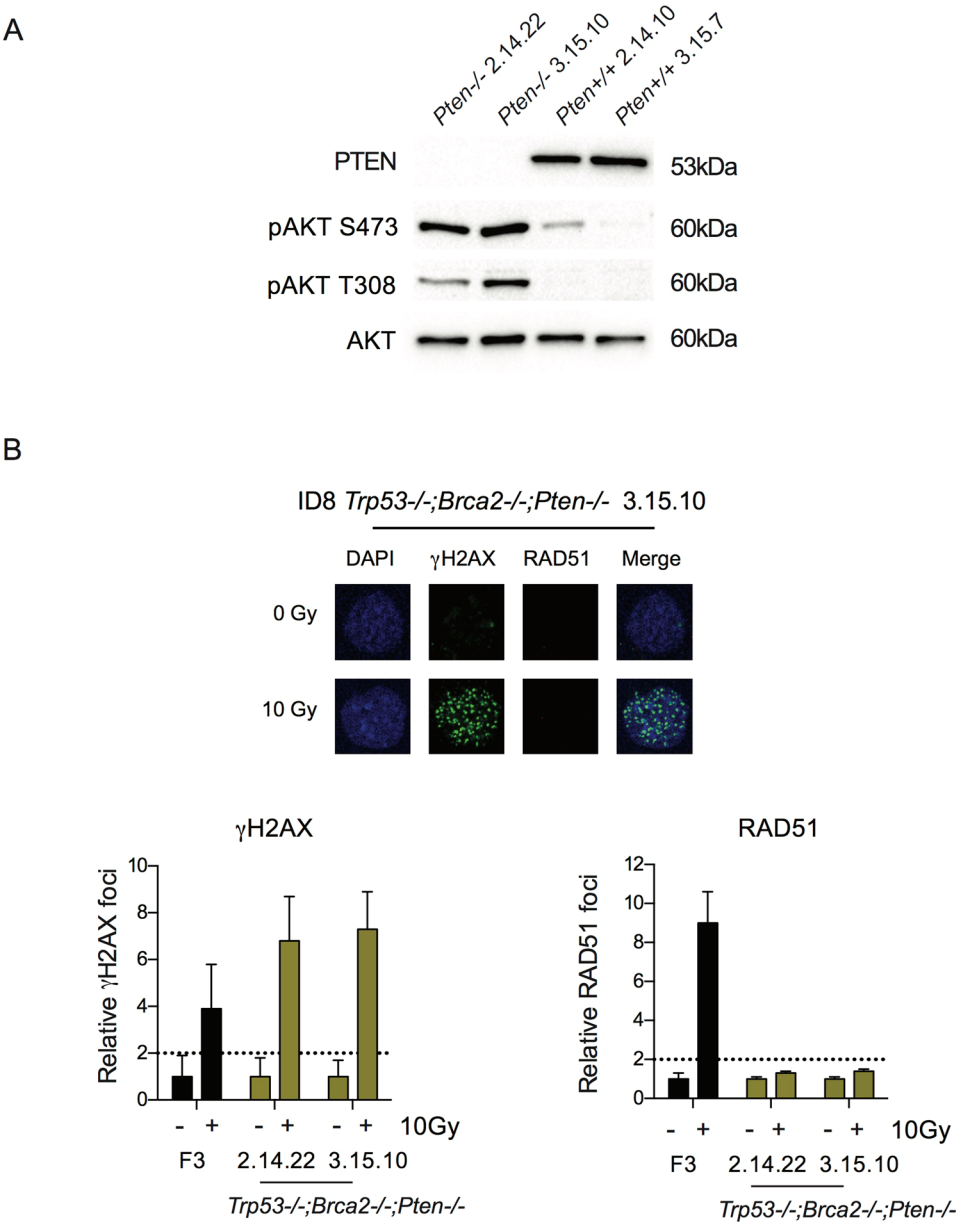
Generation of triple-deleted *Trp53* ^−/−^;*Brca2* ^−/−^;*Pten* ^−/−^ ID8 cells. (**A**) Immunoblot for PTEN and phospho-AKT following overnight serum starvation in clones isolated following *Pten* gRNA transfection. Clones 2.14.22 and 3.15.10, with bi-allelic *Pten* indels, showed absent PTEN expression and increased phosphorylation of AKT at both S473 and T308 compared to the two guide control clones, 2.14.10 and 3.15.7 that had no detectable change in *Pten* sequence. (**B**) ID8 *Trp53* ^−/−^ and *Trp53* ^−/−^;*Brca2* ^−/−^;*Pten* ^−/−^ cells were irradiated (10 Gy), fixed and stained for γH2AX and RAD51, and counterstained with DAPI. RAD51 foci were counted in up to 30 untreated and irradiated cells. Bars represent foci per cell (mean +/− SEM); γH2AX (left) and RAD51 (right); dotted lines represent two-fold increase in γH2AX and RAD51 foci/cell relative to untreated cells as above.

Multiple genomic abnormalities can co-exist within one HGSC tumour^5,17^. In addition, several of the GEMM require deletion of three genes for reliable tumorigenesis^11,12^. Therefore, we elected to target *Pten* in two of our previous *Trp53* ^−/−^;*Brca2* ^−/−^ clones (2.14 and 3.15^14^) to generate triple deleted lines. Two clones with bi-allelic alterations (Fig. S6) were generated (2.14.22 and 3.15.10) or no alterations (2.14.10, 3.15.7). The triple deleted clones lacked PTEN expression on immunoblot (Fig. 3A), and again showed marked increases in AKT phosphorylation at both S473 and T308 upon serum starvation (Fig. 3A). Of note, the triple-deleted cells did not generate RAD51 foci formation in response to 10 Gy irradiation, suggesting that absent PTEN expression did not alter the defective HR induced by *Brca2* loss (Fig. 3B).

### *In vivo* tumorigenesis

**Figure 4:**
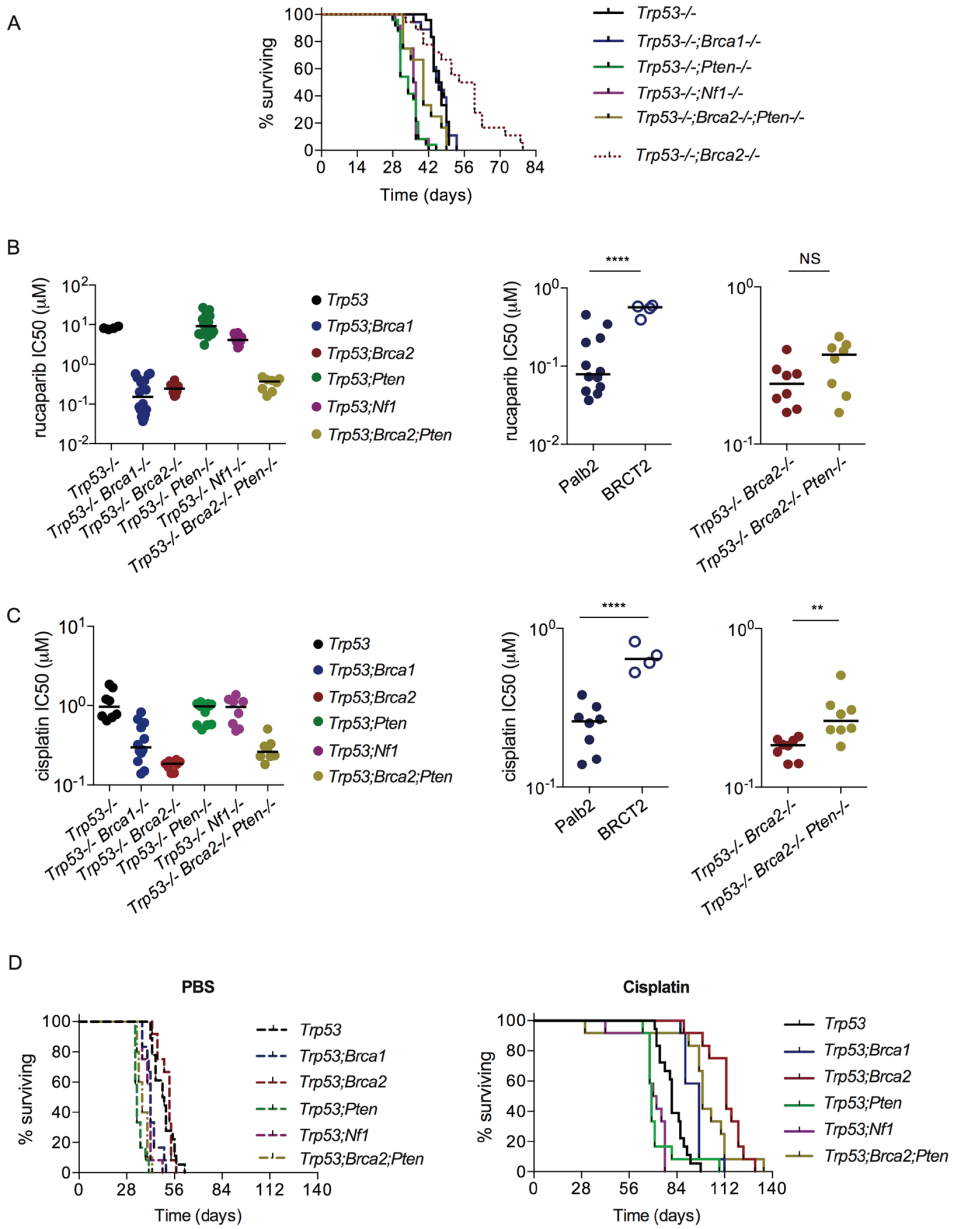
*In vivo* growth of *Trp53* ^−/−^;*Brca1* ^−/−^, *Trp53* ^−/−^;*Nf1* ^−/−^ and *Trp53* ^−/−^;*Pten1* ^−/−^ ID8 cells; platinum and PARP inhibitor sensitivity. (**A**) Cells (5 × 10^6^) were injected intraperitoneally into female C57Bl/6 mice in groups of six. Two different clones were used per genotype for *Trp53* ^−/−^;*Brca1* ^−/−^, *Trp53* ^−/−^;*Nf1* ^−/−^, *Trp53* ^−/−^;*Pten* ^−/−^ and *Trp53* ^−/−^;*Brca2* ^−/−^;*Pten* ^−/−^ tumours: a single clone (F3) was used for *Trp53* ^−/−−/−^ data. Mice were killed when they reached humane endpoints. The data for *Trp53* ^−/−^;*Brca2* ^−/−^ have been published previously^14^ but are presented here for illustrative purposes. (**B**) Cell sensitivity to PARP inhibition. At least two clones were used per genotype, except *Trp53* ^−/−^, where a single clone (F3) was used. Each dot represents one triplicate experiment. Bars represent median. Right; results for *Trp53* ^−/−^;*Brca1* ^−/−^ by site of mutation - exon 12 (Palb2 domain - clones 1.26, 1.36) and exon 19 (BRCT-2 domain - clone 6.20), and *Trp53* ^−/−^;*Brca2* ^−/−^ and *Trp53* ^−/−^;*Brca2* ^−/−^;*Pten* ^−/−^ cells. ****p < 0.0001, **p < 0.01. (**C**) Cell sensitivity to cisplatin. At least two clones were used per genotype, except *Trp53* ^−/−^, where the F3 clone was used. Each dot represents one triplicate experiment. Bars represent median. Right; results for *Trp53* ^−/−^;*Brca1* ^−/−^ by site of mutation - exon 12 (Palb2 domain - clones 1.26, 1.36) and exon 19 (BRCT-2 domain - clone 6.20) – and for *Trp53* ^−/−^;*Brca2* ^−/−^ and *Trp53* ^−/−^;*Brca2* ^−/−^;*Pten* ^−/−^ cells. ****p < 0.0001. (**D**) Cells (5 × 10^6^) were injected intraperitoneally into female C57Bl/6 mice in groups of six. Two different clones were used per genotype for *Trp53* ^−/−^;*Brca1* ^−/−^, *Trp53* ^−/−^;*Nf1* ^−/−^ *and Trp53* ^−/−^;*Pten1* ^−/−^ tumours: a single clone (F3) was used for *Trp53* ^−/−^ data. Mice received cisplatin (5 mg/kg) or PBS as intraperitoneal injections (200 µl) on days 28, 35 and 42. Mice were killed when they reached humane endpoints.

We then assessed intraperitoneal growth in female C57Bl/6 mice using at least two separate clones for each new genotype (Fig. 4A). Previously we showed that loss of p53 function significantly increased the rate of growth of intraperitoneal ID8 compared to parental controls, whilst additional loss of BRCA2 expression slowed growth relative to p53 loss alone. Results here showed that there was no difference in survival for mice bearing *Trp53* ^−/−^;*Brca1* ^−/−^ tumours compared to *Trp53* ^−/−^ (median time to reach humane endpoint 46 and 47 days respectively, p = NS), but deletion of either *Pten* or *Nf1* expression significantly accelerated growth compared to p53 loss alone (*Trp53* ^−/−^;*Pten* ^−/−^ 34 days, p < 0.0001; *Trp53* ^−/−^;*Nf1* ^−/−^ 36.5 days, p < 0.0001). Survival of mice bearing heterozygote *Trp53* ^−/−^;*Pten* ^*+/−*^ tumours (40.5 days) lay between that of *Trp53* ^−/−^ and *Trp53* ^−/−^;*Pten* ^−/−^, although differences were not significant (Fig. S7). The triple-deleted tumours showed accelerated growth (median time to reach humane endpoint 40 days) compared to both *Trp53* ^−/−^ (p = 0.003) and *Trp53* ^−/−^;*Brca2* ^−/−^ p = 0.0002), but slower than *Trp53* ^−/−^;*Pten* ^−/−^ (p = 0.0024).

### Platinum and PARP inhibitor sensitivity

We then investigated the effects of specific mutations in the ID8 cells upon sensitivity to the PARP inhibitor rucaparib *in vitro*, and to platinum chemotherapy both *in vitro* and *in vivo*. Both *Trp53* ^−/−^;*Brca1* ^−/−^ and *Trp53* ^−/−^;*Brca2* ^−/−^ cells were significantly more sensitive to rucaparib than F3 *Trp53* ^−/−^ (Fig. 4B, Table 1), whilst loss of *Pten* and *Nf1* function individually had no effect on rucaparib sensitivity. There was no overall difference between the sensitivity of *Trp53* ^−/−−/−^;*Brca1* ^−/−^ and *Trp53* ^−/−^;*Brca2* ^−/−^ cells. However, the *Brca1* BRCT-2 domain clone (6.20) was significantly less sensitive than both *Brca1* Palb2-binding domain mutants (1.26, 1.36) and the *Trp53* ^−/−^;*Brca2* ^−/−^ cells, which also have a deletion in the Palb2-binding domain (Fig. 4B). Loss of PTEN expression did not have a significant impact upon rucaparib sensitivity in the *Trp53* ^−/−^;*Brca2* ^*–/−*^;*Pten* ^−/−^ cells compared to *Trp53* ^−/−^;*Brca2* ^−/−^ (Fig. 4B).

**Table 1:** Summary of the sensitivity of ID8 clones to rucaparib and platinum.

Genotype	Rucaparib IC50 (µM) mean +/− sd	p=	Cisplatin IC50 (µM) mean +/− sd	p=
*Trp53* ^−/−^	8.23 +/− 0.62	—	1.10 +/− 0.47	—
*Trp53* ^−/−^;*Brca1* ^−/−^	0.24 +/− 0.21	<0.01	0.38 +/− 0.22	<0.0001
*Trp53* ^−/−^;*Brca2* ^−/−^	0.25 +/− 0.08	<0.01	0.18 +/− 0.03	<0.0001
*Trp53* ^−/−^;*Pten* ^−/−^	11.2 +/− 6.94	NS	0.89 +/− 0.21	NS
*Trp53* ^−/−^;*Nf1* ^−/−^	4.31 +/− 1.33	NS	0.90 +/− 0.35	NS
*Trp53* ^−/−^;*Brca2* ^−/−^;*Pten* ^−/−^	0.33 +/− 0.12	<0.01	0.28 +/− 0.10	<0.0001

Data represent IC_50_ averages from 4–8 triplicate experiments per cell line. IC_50_ values were compared using one-way ANOVA with Bonferroni’s test for multiple comparisons with *Trp53* ^−/−^ cells as comparator.

A similar pattern was seen for cisplatin *in vitro*. Both *Trp53* ^−/−^;*Brca1* ^−/−^ and *Trp53* ^−/−^;*Brca2* ^−/−^ cells were significantly more sensitive than F3 *Trp53* ^−/−^ cells, whilst loss of PTEN and NF1 expression again had no effect (Fig. 3C, Table 1). As with rucaparib, the Palb2-binding domain mutants in both *Brca1* and *Brca2* were more sensitive than the *Brca1* BRCT-2 mutant (Fig. 4C). Interestingly, there was a significant reduction in cisplatin sensitivity in the triple deleted *Trp53* ^−/−^;*Brca2* ^−/−^;*Pten* ^−/−^ cells compared to *Trp53* ^−/−^;*Brca2* ^−/−^ (Fig. 4C).

*In vivo*, there was a wide variation in survival following three doses of intraperitoneal cisplatin (5 mg/kg on days 28, 35 and 42 only). Mice bearing control *Trp53* ^*−/−*^ tumours took a median of 81 days to reach humane endpoints (Fig. 4D, Table 2). *Trp53* ^−/−^;*Pten* ^−/−^ and *Trp53* ^−/−^;*Nf1* ^−/−^ tumours produced the worst survival (median 69 and 71 days respectively; p < 0.0001 and p = 0.0001 respectively compared to *Trp53* ^−/−^). For *Trp53* ^−/−^;*Brca1* ^−/−^, survival was extended to 97 days (p = 0.0003 compared to *Trp53* ^−/−^), whilst the longest survival was seen with *Trp53* ^−/−^;*Brca2* ^−/−^ tumours (median 113 days), which was significantly longer than both *Trp53* ^−/−^ and *Trp53* ^−/−^;*Brca1* ^−/−^ (p < 0.0001 for both comparisons). Mice bearing the triple-deleted *Trp53* ^−/−^;*Brca2* ^−/−^;*Pten* ^−/−^ tumours survived 99 days, which was significantly more than *Trp53* ^−/−^ (p < 0.0001; Table 2) but less than *Trp53* ^−/−^;*Brca2* ^−/−^, although this latter comparison did not reach statistical significance (p = 0.078). In the *Trp53* ^−/−^;*Brca1* ^−/−^ experiments, median survival for the PALB2 mutant (clone 1.36) was longer (97 days) than for the BRCT-2 mutant (clone 6.20; 89 days), although again this did not reach statistical significance (p = 0.066) (Fig. S8).

**Table 2:** Summary of survival following cisplatin treatment *in vivo*.

Genotype	Median survival cisplatin (days)	Hazard ratio (log-rank)	p=
*Trp53* ^−/−^	81	—	—
*Trp53* ^−/−^;*Brca1* ^−/−^	97	0.34	0.0003
*Trp53* ^−/−^;*Brca2* ^−/−^	113	0.23	<0.0001
*Trp53* ^−/−^;*Pten* ^−/−^	69	4.07	<0.0001
*Trp53* ^−/−^;*Nf1* ^−/−^	71	3.19	0.0001
*Trp53* ^−/−^;*Brca2* ^−/−^;*Pten* ^−/−^	99	0.29	<0.0001

Data represent median survival (time to reach humane endpoint) for mice bearing ID8 clones treated with cisplatin (5 mg/kg) on days 28, 35 and 42. Data were compared using log-rank test.

### Trp53 knockout in Dicer^−/−^;Pten^−/−^ DKO cells

**Figure 5:**
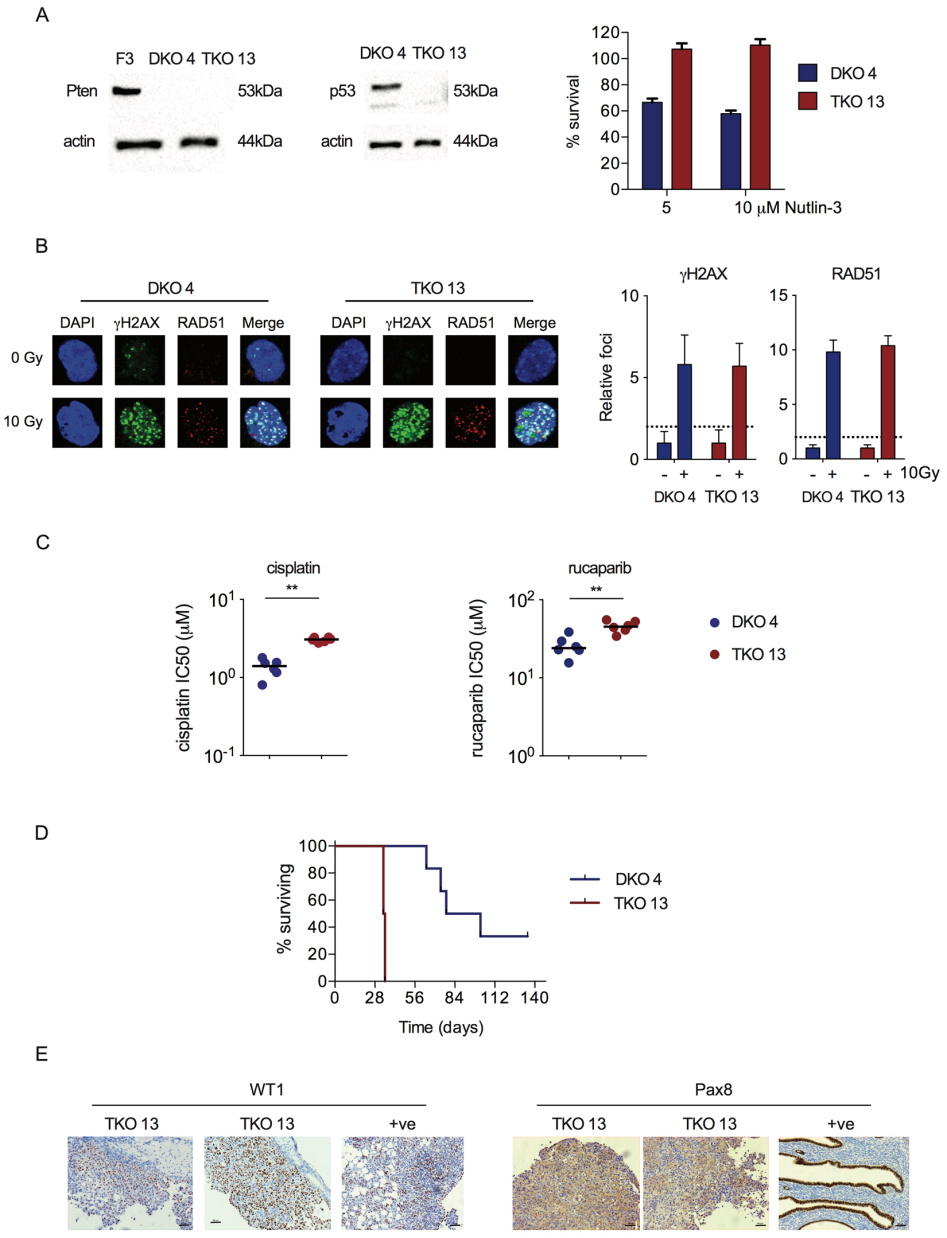
Generation and evaluation of *Dicer* ^−/−^;*Pten* ^−/−^;*Trp53* ^−/−^ TKO cells. (**A**) OvidT 497 *Dicer* ^−/−^;*Pten* ^−/−^ (DKO) cells were transfected with PX459 encoding *Trp53* gRNA. Clone 4 contained no *Trp53* mutation; clone 13 (TKO) contained bi-allelic *Trp53* exon 5 mutations. Expression of PTEN and p53 was assessed by immunoblot (left). F3 = ID8 *Trp53* ^−/−^. Sensitivity to Nutlin-3 was assessed by MTT assay (right). (**B**) Homologous recombination was assessed in DKO 4 and TKO 13 cells as previously. (**C**) Sensitivity of DKO 4 and TKO 13 cells to cisplatin. Each dot represents one triplicate experiment. Bars represent median. *p < 0.01. (**D**) Cells (5 × 10^6^) were injected intraperitoneally into female C57Bl/6 mice in groups of six. Mice were killed when they reached humane endpoints. Excised tumours were fixed in formalin and stained for WT1 and PAX8. Each TKO 13 section comes from a separate mouse. Positive controls (+ve) are ID8 tumour (WT1) and normal mouse fallopian tube (PAX8), both from^14^. Bars represent 50 µm.

Finally, we targeted *Trp53* in OvidT 497 *Dicer* ^−/−^;*Pten* ^−/−^ DKO fallopian tube carcinoma cells using the same guide RNA used to generate ID8 *Trp53* ^−/−^ clone F3. Triple knockout (TKO; *Dicer* ^−/−^;*Pten* ^−/−^;*Trp53* ^−/−^) clone 13 had a biallelic deletion in exon 5 (Fig. S8), with absent p53 on immunoblot, and increased resistance to Nutlin-3 compared to cells from a DKO control clone (clone 4) that had been transfected with the *Trp53* gRNA but contained no *Trp53* mutation (Fig. 5A). Both DKO and TKO cells retained functional HR (Fig. 5B). However, interestingly, *Trp53* loss induced small but statistically significant changes in sensitivity to both cisplatin and rucaparib in TKO cells *in vitro* (Fig. 5C). Loss of p53 function produced a highly significant reduction in time to reach humane endpoints - median survival (Fig. 5D; median 34.5 days vs 90 days; p < 0.0001). *In vivo*, TKO tumours demonstrated nuclear WT1 staining (Fig. 5E). However, there was only weak cytoplasmic PAX8 staining, in contrast to strong nuclear staining seen in fallopian tube epithelial cells (Fig. 5E).

## Discussion

Here we have extended our previous results, and generated further derivatives of the ID8 murine ovarian carcinoma model using CRISPR/Cas9 gene editing. Using one of our previous *Trp53* ^−/−^ clones, we have generated further double mutants, with deletions in *Brca1*, *Pten* and *Nf1* in addition to loss *Trp53*, as well as triple mutants lacking *Trp53*, *Brca2* and *Pten*. We have also generated a *Trp53* mutant derivative of a transplantable murine fallopian tube carcinoma cell line. Collectively, our results indicate that loss of genes known to be mutated or deleted in HGSC can alter intraperitoneal tumour growth and response to therapy. Moreover, the responses to therapy mirror those seen in patients, reinforcing the utility of these models.

As expected, we show that loss of BRCA1 or BRCA2 function induces defective homologous recombination and sensitises cells to both platinum and PARP inhibitor therapy. There was no overall difference in rucaparib sensitivity between *Brca1* ^−/−^ or *Brca2* ^−/−^ cells, which accords with results from part 1 of the ARIEL2 trial of rucaparib, in which radiological response rates were almost identical in *BRCA1* (79%) and *BRCA2*-mutated (82%) tumours^8^. However, our data show that mice bearing *Trp53* ^−/−^;*Brca2* ^−/−^ tumours have the longest survival following platinum treatment. It is now clear that patients with germline *BRCA2* mutations have significantly greater long term survival than patients without mutations and even those with germline *BRCA1* mutations^6^, which may reflect extreme sensitivity to platinum-based chemotherapy.

Our results suggest that the location of the mutation within *Brca1* might influence sensitivity - the two clones with mutations in the PALB2-binding domain were significantly more sensitive to both cisplatin and rucaparib *in vitro* than the BRCT-2 domain mutant, although the difference in survival following *in vivo* platinum treatment was not significant (p = 0.066). In patients with HGSC, it remains unclear whether the type and location of mutations within *BRCA1* and *BRCA2* influence response to treatment or overall survival. The Australian Ovarian Cancer Study found no overall effect of location within the gene, nor of mutation type, on relapse or survival^18^. However, the sample size (n = 134 mutation carriers) was possibly too small to allow enrichment of mutations in one specific region. A retrospective analysis of 445 women with germline *BRCA1/2* mutations (79% of whom had had breast and/or ovarian cancer) found a non-significant (p = 0.06) reduction in overall survival in those with *BRCA1* exon 20 mutations compared to those with mutations in *BRCA1* exons 2 or 11^19^. However, again the number of cases with mutation in a specific exon was low. By contrast, in a transgenic murine breast cancer model, nonsynonymous missense mutation in the *Brca1* RING domain (C61G) was associated with poorer response to cisplatin and PARP inhibitor therapy than a complete *Brca1* knockout, despite similar tumour morphology and copy number alterations^20^. Large consortia and meta-analyses will help to address genotype/phenotype relationships, especially relating to survival, but our cells may help to answer specific questions relating to protein function and chemotherapy sensitivity, and CRISPR/Cas9 technology allows for further exon-specific deletions to be created.

One striking feature of our results was the increased rate of intra-tumoural growth and reduced survival following platinum treatment of tumours with double *Trp53*;*Pten* or *Trp53*;*Nf1* mutations compared to those with *Trp53* mutations alone. Moreover, additional loss of PTEN expression significantly accelerated the growth of *Trp53* ^−/−^;*Brca2* ^−/−^ tumours, and significantly reduced platinum sensitivity *in vitro*. There are few clear prognostic data on the influence of NF1 loss on HGSC patient survival, but our results correlate with data showing that loss of PTEN expression is associated with poor outcome^9^. We also show clearly that loss of PTEN expression alone does not induce defective homologous recombination in ID8 cells, although the additional loss of PTEN does not abrogate defective homologous recombination or PARP inhibitor sensitivity seen in *Trp53* ^−/−^;*Brca2* ^−/−^ cells. We found OvidT 497 *Dicer* ^−/−^;*Pten* ^−/−^ DKO cells to be HR competent, as defined by radiation-induced RAD51 foci formation, and that loss of p53 function in the *Dicer* ^−/−^;*Pten* ^−/−^;*Trp53* ^−/−^ TKO cells induced a degree of both platinum and PARP inhibitor resistance. This contrasts with data from HCT116 cells^21^ and from endometrioid endometrial carcinoma^22^, whereby loss of PTEN was associated with partial loss of HR and sensitivity to PARP inhibition. In PTEN-deficient prostate carcinoma cells, treatment with a PARP inhibitor induced apoptosis but only in the absence of p53^23^. Overall, these data imply that the relationship between PTEN, p53 and DNA double strand break repair is complex and varies by tumour type; however, our results do suggest that classification of PTEN-mutant HGSC as HR defective in the absence of mutations in *BRCA1* or *BRCA2* ^4^ may be erroneous.

The *Dicer* ^−/−^;*Pten* ^−/−^;*Trp53* ^−/−^ TKO cells also demonstrate clearly that loss of p53 function significantly enhances intra-peritoneal tumour growth. This is in keeping with our original ID8 work^14^ and data from the *Dicer* ^−/−^;*Pten* ^−/−^;*Trp53* ^*LSL-R172H/*+^ TKO mice, in which expression of mutant p53 resulted in a more aggressive phenotype than *Dicer* ^−/−^;*Pten* ^−/−^ alone^24^. Unfortunately, no transplantable cell lines have been described from the *Dicer* ^−/−^;*Pten* ^−/−^;*Trp53* ^*LSL-R172H/*+^ TKO mice with which to allow comparison of tumours lacking p53 expression with those harbouring a potential gain-of-function point mutation. One potential criticism of the ID8 model is that it is likely to be of ovarian origin, whereas the majority of HGSC cases arise from precursor lesions within the distal fallopian tube^25,26^. The *Dicer* ^−/−^;*Pten* ^−/−^ DKO represents a novel model of tubal carcinogenesis, although the exact cell of origin remains unclear^15^. Our immunohistochemistry show the DKO and TKO tumours to express WT1 but not PAX8, suggesting that they have either lost PAX8 expression during tumorigenesis, or may not arise in the secretory cells of the tubal fimbria– interestingly, in transgenic *Pten* ^−/−^;*Trp53* ^*LSL-R172H/*+^ mice, tumours were still able to form following removal of the fallopian tumours, suggesting that HGSC can arise in the ovaries^24^.

In conclusion, we have generated new transplantable murine models that recreate further key mutations of HGSC. These cell lines will be powerful tools, alongside transgenic models and primary patient material, to elucidate HGSC biology and chemotherapy resistance, and all of the ID8 lines are freely available to other researchers upon request.

## Materials and Methods

### Cells

The production of ID8 *Trp53* ^−/−^ cells was described previously^14^. OvidT 479 cells were isolated from tumours arising in *Dicer-Pten* double knockout (DKO) mice (*Amhr2* ^*cre/*+^ *Dicer* ^*flox/flox*^ *Pten* ^*flox/flox*^) as previously described^15,27^.

### CRISPR/Cas9 and selection

Guide RNAs targeting the PALB2-binding domain in exon 12 and the BRCT-2 domain in exon 19 of *Brca1* were designed using two open-access software programs, CHOPCHOP (https://chopchop.rc.fas.harvard.edu/) and CRISPR design (http://crispr.mit.edu/), and ligated into BbsI-linearized pSpCas9(BB)-2A-Puro [PX459^28^, a gift from Feng Zhang via Addgene]. The gRNA for *Pten* was a gift from Douglas Strathdee (CRUK Beatson Institute, Glasgow, UK) and targeted exon 5, encoding the phosphatase domain. The gRNA for *Nf1* was designed to target exon 2 as previously described^29^. gRNAs for *Pten* and *Nf1* were ligated into PX330^30^ (a gift from Walter Jackson via Addgene, ref 78621).

Cells were transfected as previously^14^, omitting puromycin selection for *Pten* and *Nf1* targeting. PCR primers spanning target sites of deletion are listed in Table S1. PCR products were cloned using InFusion kit (ClonTech) and clones with large PCR deletions were selected for subsequent analysis. Remaining clones were screened using the Surveyor Nuclease Assay (Integrated DNA Technology). Mutations were confirmed by Sanger sequencing. All sequence alignment was performed using MAFFT version 7 (http://mafft.cbrc.jp/).

### γH2AX/Rad51 assay

24 h treatment following treatment with 10 µM rucaparib (Clovis Oncology, Boulder, CO), or following 10 Gy irradiation, cells were fixed and stained for γH2AX and Rad51 foci as previously^14^ using a Zeiss 710 confocal microscope and Zen software (Zeiss). Microscope settings were as follows: 20x objective, 1024 × 1024 frame, 6.25 s scan time. Lasers used were 561 nm (red), 488 nm (green) and 405 nm (DAPI). Fluorophores were ALEXA-568 (for RAD51) and ALEXA-488 (for γH2AX). Foci were counted in at least 30 nuclei per condition without any image manipulation. Images presented in figures were captured from raw Zen files using Photoshop CS5 v12.1 software (Abode), and processed in their entirety using the ‘AutoContrast’ tool, and then cropped to show single representative cells. Classification of homologous recombination competence was performed as previously^16^.

### Immunoblot and co-immunoprecipitation

For detection of phospho-AKT, cells were serum-starved for 16 hours prior to lysis. Antibodies used were: phospho-AKT (T308) (Cell Signalling 13038), phospho-AKT (S473) (Cell Signaling 40600), AKT (Cell Signalling 4685) and PTEN (Cell Signalling 9552). Co-immunoprecipitation of Ras-GTP was performed using Ras pulldown activation assay kit (BK008-S; Cytoskeleton, Denver CO, USA). 5 × 10^5^ cells were plated on 10 cm dishes for 72 hours, then serum-starved for 16 hours prior to lysis. All subsequent steps were performed according to manufacturer’s instructions. The same lysates were probed for phospho-ERK (Cell Signalling 4695) and total ERK.

All immunoblot images were acquired using a BioRad ChemiDoc MP imaging system and Image Lab 5.0 software (both BioRad, Watford UK). Images were visualised using Photoshop CS5 v12.1 software (Abode), and processed in their entirety using the ‘AutoContrast’ tool, and then cropped to include relevant lanes only. All immunoblot images presented derive from single blots and no Photoshop touch-up tools were used in any image. All loading controls come from blots stripped and re-probed.

### *In vitro* cytotoxicity

ID8 cells (3 × 10^4^ cells/well of a 24 well plate) were treated with 3 nM–30 μM rucaparib (Clovis Oncology, Boulder CO, USA) or 10 nM–1000 μM cisplatin (Accord Healthcare, Harrow, UK via Beatson West of Scotland Cancer Centre chemotherapy pharmacy) 4 hours after initial plating. 68 hours thereafter, cell survival was assessed using sulphorhodamine B (rucaparib) or MTT^31^ (cisplatin) assay.

### *In vivo* experiments and immunohistochemistry

All experiments complied with the UK welfare guidelines^32^ and were conducted under specific personal and project license authority (70/8645) with ethical approval (University of Glasgow) in dedicated animal facilities. 5 × 10^6^ cells were inoculated intraperitoneally (IP) in 6–8 week old female C57Bl/6 mice (Charles River Laboratories, UK) in groups of 6. A minimum of two clones per genotype were used in all experiments. Mice were monitored regularly and killed upon reaching UK Home Office limits. All decisions about animal welfare and experiment endpoints were made by D.A. or S.M. independently of main study investigators to prevent bias. Cisplatin (5 mg/kg in 200 µl PBS) or PBS was administered intraperitoneally on days 28, 35 and 42 only. Ascites was collected and all visible tumour deposits dissected out, and either snap frozen or fixed in neutral-buffered 4% paraformaldehyde. 5 µm sections from formalin-fixed paraffin-embedded tumours were stained (Dako Autostainer, Dako, UK). The following antibodies were used for immunohistochemistry - WT1 (Can (R9), Abcam, 1:250); PAX8 (ZR-1, Zeta, 1:30). Images were captured on a Zeiss Observer Z1 microscope (20x objective) using Zen software (Zeiss), visualised using Photoshop CS5 v12.1 software (Abode), and processed in their entirety using the ‘AutoContrast’ tool.

### Statistics

All analyses were performed using Prism v6.0 (Graphpad, CA). One-way ANOVA with Bonferroni’s test for multiple comparisons and unpaired *t*-test were used to compare IC_50_ values. Mouse survival was compared using the log-rank test. p < 0.05 was considered significant.

## Electronic supplementary material

Supplementary information
