# Author Correction: CRISPR/Cas9-derived models of ovarian high grade serous carcinoma targeting *Brca1*, *Pten* and *Nf1*, and correlation with platinum sensitivity

**DOI:** 10.1038/s41598-018-24099-3

**Published:** 2018-04-13

**Authors:** Josephine B. Walton, Malcolm Farquharson, Susan Mason, Jennifer Port, Bjorn Kruspig, Suzanne Dowson, David Stevenson, Daniel Murphy, Martin Matzuk, Jaeyeon Kim, Seth Coffelt, Karen Blyth, Iain A. McNeish

**Affiliations:** 1 Institute of Cancer Sciences, University of Glasgow, Glasgow, UK; 2 Cancer Research UK Beatson Institute, Glasgow, UK; 3 Department of Pathology and Immunology, Baylor College of Medicine, Houston, TX USA; 4 Departments of Biochemistry and Molecular Biology, Indiana University School of Medicine, Indianapolis, IN USA

Correction to: *Scientific Reports* 10.1038/s41598-017-17119-1, published online 04 December 2017

This Article contains errors.

In Figure 5A, the actin loading control western blot is missing a lane. The correct Figure 5 appears below as Figure 1.

**Figure 1:**
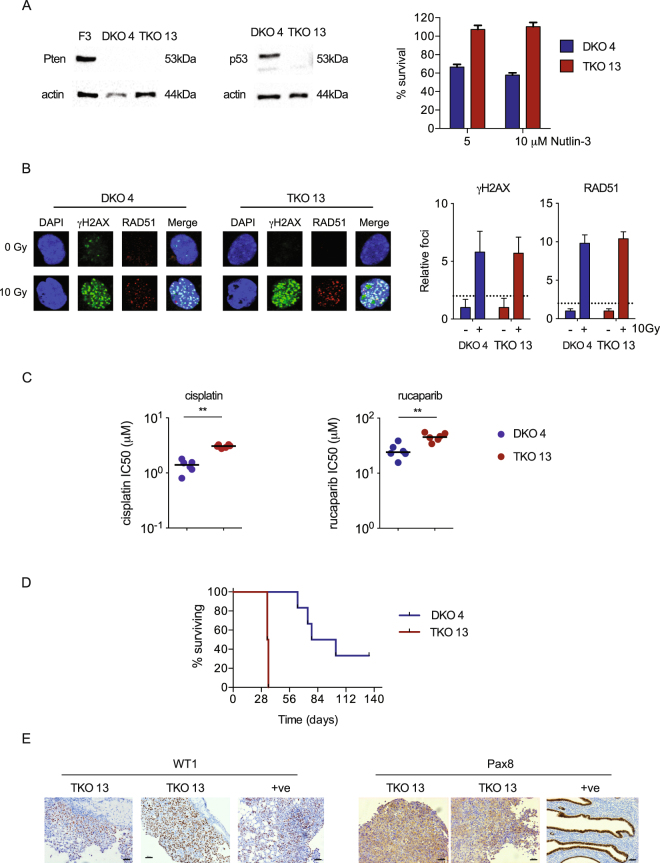
Generation and evaluation of *Dicer*^−/−^; *Pten*^−/−^; *Trp53*^−/−^ TKO cells. (**A**) OvidT 497 *Dicer*^−/−^; *Pten*^−/−^ (DKO) cells were transfected with PX459 encoding *Trp53* gRNA. Clone 4 contained no *Trp53* mutation; clone 13 (TKO) contained bi-allelic *Trp53* exon 5 mutations. Expression of PTEN and p53 was assessed by immunoblot (left). F3 = ID8 *Trp53*^−/−^. Sensitivity to Nutlin-3 was assessed by MTT assay (right). (**B**) Homologous recombination was assessed in DKO 4 and TKO 13 cells as previously. (**C**) Sensitivity of DKO 4 and TKO 13 cells to cisplatin. Each dot represents one triplicate experiment. Bars represent median. *p < 0.01. (**D**) Cells (5 × 106) were injected intraperitoneally into female C57Bl/6 mice in groups of six. Mice were killed when they reached humane endpoints. Excised tumours were fixed in formalin and stained for WT1 and PAX8. Each TKO 13 section comes from a separate mouse. Positive controls (+ve) are ID8 tumour (WT1) and normal mouse fallopian tube (PAX8), both from^14^. Bars represent 50 μm.

In addition, in the Results section under the sub-heading ‘Platinum and PARP inhibitor sensitivity’,

“There was no overall difference between the sensitivity of *Trp53*^−/−−/−^;*Brca1*^−/−^ and *Trp53*^−/−^;*Brca2*^−/−^ cells.”

should read:

“There was no overall difference between the sensitivity of *Trp53*^−/−^;*Brca1*^−/−^ and *Trp53*^−/−^;*Brca2*^−/−^ cells.”

Finally, in the Discussion section,

“Using one of our previous *Trp53*^−/−^ clones, we have generated further double mutants, with deletions in *Brca1*, *Pten* and *Nf1* in addition to loss *Trp53*, as well as triple mutants lacking *Trp53*, *Brca2* and *Pten*.”

should read:

“Using one of our previous *Trp53*^−/−^ clones, we have generated further double mutants, with deletions in *Brca1*, *Pten* and *Nf1* in addition to loss of *Trp53*, as well as triple mutants lacking *Trp53*, *Brca2* and *Pten*.”

