# Supplementary material for: CRISPR/Cas9-derived models of ovarian high grade serous carcinoma targeting Brca1, Pten and Nf1, and correlation with platinum sensitivity
Source: Sci Rep. 2017 Dec 4;7:16827. doi: 10.1038/s41598-017-17119-1 (PMC5715106; doi:10.1038/s41598-017-17119-1)

CRISPR/Cas9-derived models of ovarian high grade serous carcinoma targeting *Brca1, Pten* and *Nf1*, and correlation with platinum sensitivity

J.B. Walton[1], M. Farquharson[1], S. Mason[2], J. Port[2], B. Kruspig[2], S. Dowson[1], D. Stevenson[2], D. Murphy[1,2], M. Matzuk[3], J. Kim[4], S. Coffelt[2], K. Blyth[2], I.A. McNeish[1]*

Supplementary information

Supplementary table and figure legends

Table S1

PCR primers used for specific genes and guides

Figure S1.

Sanger sequencing of the region around exon 12 (upper) and exon 19 (lower) of *Brca1* in the three *Trp53*$^{-/-}$;*Brca1*$^{-/-}$ clones 1.26, 1.36 and 6.20. Guide RNA sequences used to generate each clone are shown in green.

Figure S2.

ID8 *Trp53*$^{-/-}$ and *Trp53*$^{-/-}$;*Brca1*$^{-/-}$ cells were treated with rucaparib (10 μM) for 24h, fixed and stained for γH2AX and RAD51, and counterstained with DAPI. RAD51 foci were counted in up to 30 untreated and irradiated cells. Bars represent mean (+/- SEM) γH2AX (left) and RAD51 (right) foci per cell; dotted lines represents two-fold increase in γH2AX and RAD51 foci/cell relative to untreated cells, suggestive of induction of DNA double strand breaks and functional homologous recombination respectively{Mukhopadhyay, 2010 #3128}.

Figure S3.

Sanger sequencing of the region around exon 5 of *Pten* in the four *Trp53*$^{-/-}$;*Pten*$^{-/-}$ clones 1.11, 1.12, 1.14 and 1.15. Guide RNA sequence used to generate the clones is shown in green.

Figure S4.

Sanger sequencing of the region around exon 2 of *Nf1* in the two *Trp53*$^{-/-}$;*Nf1*$^{-/-}$ clones 1.20, and 1.23. Guide RNA sequence used to generate the clones is shown in green.

Figure S5.

Confocal microscopy images γH2AX and RAD51 staining of *Trp53*$^{-/-}$;*Pten*$^{-/-}$,*Trp53*$^{-/-}$;*Nf1*$^{-/-}$ and *Trp53*$^{-/-}$;*Pten*$^{+/-}$ cells following 10Gy irradiation. See Fig. 2F for quantification.

Figure S6.

Sanger sequencing of the region around exon 5 of *Pten* in the two *Trp53*$^{-/-}$;*Brca2*$^{-/-}$;*Pten*$^{-/-}$ clones 2.14.22 and 3.15.10. Guide RNA sequence used to generate the clones is shown in green.

Figure S7.

5x10^{6} *Trp53*$^{-/-}$;*Pten1*$^{+/-}$ cells were injected intraperitoneally into female C57Bl/6 mice in groups of six. Two different clones were used. Mice were killed when they reached humane endpoints.

Figure S8.

Results from Fig. 4D for *Trp53*$^{-/-}$;*Brca1*$^{-/-}$ tumours treated with intraperitoneal cisplatin with data for clones 1.36 and 6.20 displayed separately.

Table S1

Gene	Forward	Reverse
Brca1 Guide 1	5′-TTATCTCACAGTAGCGAGTC-3	5′-GTCCATAGCATCTCCTTCTA-3′
Brca1 Guide 6	5′-TGCTAATCTGTCAAGGAACC-3′	5′-CCATCAGCTAGGATCTAAACC-3′
Pten	5′-TACTTGACAAGAGGCCTGGG-3′	5′-GGTCCACAAAGAGGGAGGAA-3′
Nf1	5′-GGAAAACACTGGATAGAAGATTTG-3′	5′-GCTTCAACGGGAATAAAAACCTG-3′

Supplementary Figures

S1

Brca1_1.26 1.36

```
                  GCAGCAGGAAATGGCT-CACC Guide1
GTATAACCTGATAAAGCTGCAGCAGGAAATGGCT-CACCTGGAAGCTGTGCTGGAGCAG Brca1_Palb2
GTATAACCTGATAAAGCTGCAGCAGGAAATGG---CACCTGGAAGCTGTGCTGGAGCAG JW01_1.26
GTATAACCTGATAAAGCTGCAG-------------CACCTGGAAGCTGTGCTGGAGCAG JW02_1.26
GTATAACCTGATAAAGCTGCAGCAGGAAATGGCTCCACCTGGAAGCTGTGCTGGAGCAG JW02_1.36
GTATAACCTGATAAAGCTGCAGCAGGAAATG--------TGGAAGCTGTGCTGGAGCAG JW03_1.36
```

Brca1_6.20

```
GGTTTTATTCCAGCAGCCTCATGGGTTCTCACAGCTAATTTTTAACTCCAGGTCCAAGACATATATATATATGGTACATATAACATACATT
GGTTTTATTCCAGCAGCCTCATGGGTTCTCACAGCTAAT----------------------------------------------------
GGTTTTATTCCAGCAGCCTCATGGGTTCTCACAGCTAA-----------------------------------------------------

TAGGCAAAATACTCATACATAAAATAAAAAGAATGAATGTTTAAAATATATTTTCTAAGATAGGGTCTTACCTGGGTTTGGTGGCATGTCT
-------------------------------------------------------------------------------------------
-------------------------------------------------------------------------------------------

CTTTAATCTCAGCTCTTGGGAGAAGGCAGAGTCAGGCAGATTTCTGCATCCCAGGCCAGCCAGGCTCCACGGTCTTGTCTCCAAAAAATAA
-------------------------------------------------------------------------------------------
-------------------------------------------------------------------------------------------

CAAAGACAAGGTTTCATTATGTAATGCTGGCTGGCCTGGACATGAGTGTAGTGCCTTAATGCTTCGTGGCTGGGCCGTCACCCACCCCAGT
-------------------------------------------------------------------------------------------
-------------------------------------------------------------------------------------------

                                                                                     CGATCC
AATCACAGAGGTTTGTGTTTGGTTTCAGCATGAATTTGAAGTCAAAGGAGATGTTGTGACTGGAAGAAATCACCAAGGTCCAAGGCGATCC
-------------------------------------------------------------------------------------------
-------------------------------------------------------------------------------------------

AGAGAATCCCG Guide6
AGAGAATCCCGGGAAAAGGTAAAGTCCCTCTTTATGTGACA Brca1_BRCT2
-----------------------------CTTTATGTGACA JW02_6.20
-----------------------------CTTTATGTGACA JW03_6.20
```

S2

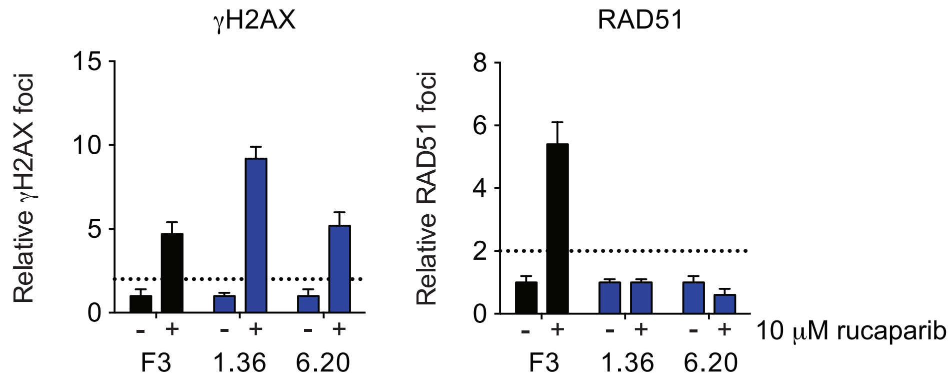

S3

Pten_1.11 1.12

```
            TGTGCATATTTATTGCATCG Guide1
GGTGTAATGATTTGTGCATATTTATTGCATCGGGGCAAATTTTTAAAGGCACAAGAGGCCCTAGATTTTTATGGGGA Pten_exon5
GGTGTAATGATTTGTGCATAT-------------------TTTTAAAGGCACAAGAGGCCCTAGATTTTTATGGGGA JW14_1.11
GGTGTAATGATTTGTGCATAT---------------------------------GAGGCCCTAGATTTTTATGGGGA JW17_1.11
GGTGTAATGATTTGTGCATATTTAT----TCGGGGCAAATTTTTAAAGGCACAAGAGGCCCTAGATTTTTATGGGGA JW25_1.12
GGTGTAATGATTTGTGCATATTTATTG--TCGGGGCAAATTTTTAAAGGCACAAGAGGCCCTAGATTTTTATGGGGA JW28_1.12
```

Pten_1.14

```
AGTTAACCTATGTTTTTAAATAAAAAATTTAAAATTTTCTTACACTGGGATTATCTTTTTGCAACAGTTGCACAGTATCCTT
AGTTAACCTATGTTTTTAAATAAAAAATTTAAAATTTTCTTACACTGGGATTATCTTTTTGCAACAGTTGCACAGTATCCTT
AGTTAACCTATG----------------------------------------------------------------------

TTGAAGACCATAACCCACCACAGCTAGAACTTATCAAACCCTTCTGTGAAGATCTTGACCAATGGCTAAGTGAAGATGACAA
TTGAAGACCATAACCCACCACAGCTAGAACTTATCAAACCCTTCTGTGAAGATCTTGACCAATGGCTAAGTGAAGATGACAA
----------------------------------------------------------------------------------

                                                       TGTGCATATTTATTGCATCG Guide1
TCATGTTGCAGCAATTCACTGTAAAGCTGGAAAGGGACGGACTGGTGTAATGATTTGTGCATATTTATTGCATCGGGGCAAA
TCATGTTGCAGCAATTCACTGTAAAGCTGGAAAGGGACGGACTGGTGTAATGATTTGT------------------------
----------------------------------------------------------------------------------

TTTTTAAAGGCACAAGAGGCCCTAGATTTTTATGGGGAAGTAAGGACCAGAGAC Pten_exon5
--------GGCACAAGAGGCCCTAGATTTTTATGGGGAAGTAAGGACCAGAGAC JW39_1.14
--------------------------TTTTTATGGGGAAGTAAGGACCAGAGAC JW44_1.14
```

Pten_1.15

```
           TGTGCATATTTATTGCATCG Guide1
GTGTAATGATTTGTGCATATTTATTGCATCGGGGCAAATTTTTAAAGG Pten_exon5
GTGTAATGATTTGTGCATATTTATTGCA--GGGGCAAATTTTTAAAGG JW53_1.15
GTGTAATGATTTGTGCATATTTATT----CGGGGCAAATTTTTAAAGG JW56_1.15
```

S4

Nf1_1.20 1.23

```
            AGTCAGCACCGAGCAC-AACAAGG Guide1
CACACATACCAAAGTCAGCACCGAGCAC-AACAAGGAGTGTCTGATCAACATTTCCAAATA Nf1_exon2
CACACATACCAAAGTCAGCACCGAGCACAAACAAGGAGTGTCTGATCAACATTTCCAAATA JW49_1.20
CACACATACCAAAGTCAGCACCGAGCA--AACAAGGAGTGTCTGATCAACATTTCCAAATA JW50_1.20
CACACATACCAAAGTCAGCACCGAGCACAAACAAGGAGTGTCTGATCAACATTTCCAAATA JW01_1.23
CACACATACCAAAGTCAGCACCGAGCACAAACAAGGAGTGTCTGATCAACATTTCCAAATA JW02_1.23
```

S5

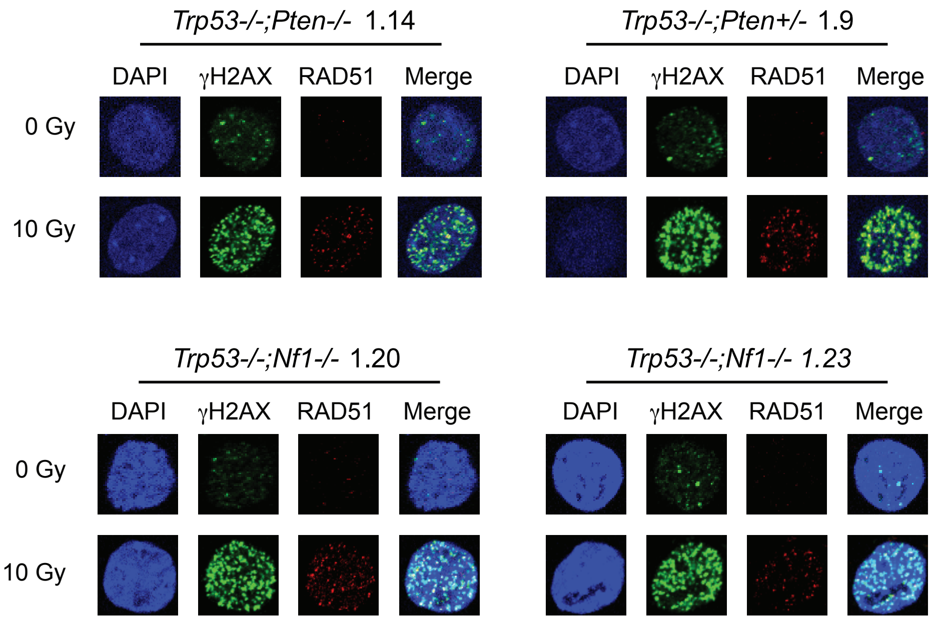

S6

```
Brca2;Pten_2.14.22 3.15.10
  TGTGCATATTTATTGC-ATCG Guide1
TTTGTGCATATTTATTGC-ATCGGGGCAAA TTTTTAAAGG CACAAGAGGC   563 Pten_exon5
TTTGTGCATA---------ATCGGGGCAAA TTTTTAAAGG CACAAGAGGC   519 JW41_2.14.22
TTT------------------GGGGCAAA TTTTTAAAGG CACAAGAGGC   508 JW42_2.14.22
TTT------------------GGGGCAAA TTTTTAAAGG CACAAGAGGC   566 JW11_3.15.10
TTTGTGCATATTTATTGCAATCGGGGCAAA TTTTTAAAGG CACAAGAGGC   589 JW12_3.15.10
```

S7

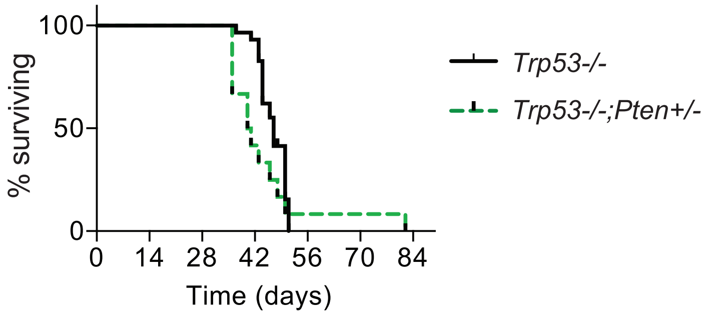

S8

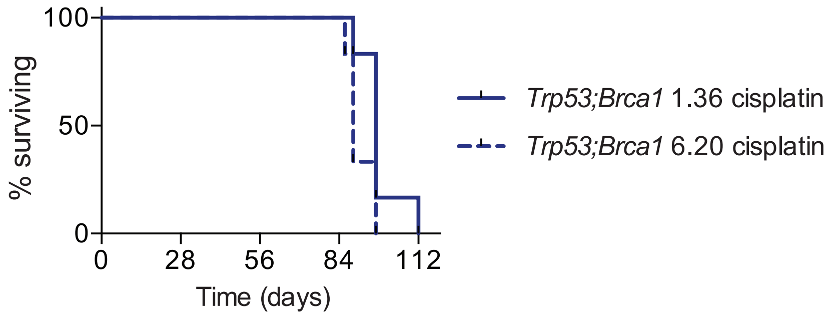

Supplement: Supplementary file 1 — Supplementary information [file 41598_2017_17119_MOESM1_ESM.pdf]
